# Cytotoxic Constituents from the Rhizomes of *Curcuma zedoaria*

**DOI:** 10.1155/2014/321943

**Published:** 2014-07-13

**Authors:** Omer Abdalla Ahmed Hamdi, Syarifah Nur Syed Abdul Rahman, Khalijah Awang, Norhanom Abdul Wahab, Chung Yeng Looi, Noel Francis Thomas, Sri Nurestri Abd Malek

**Affiliations:** ^1^Center for Natural Products and Drugs Discovery (CENAR), University of Malaya, 50603 Kuala Lumpur, Malaysia; ^2^Department of Chemistry, Faculty of Science, University of Malaya, 50603 Kuala Lumpur, Malaysia; ^3^Institute of Biological Sciences, Faculty of Science, University of Malaya, 50603 Kuala Lumpur, Malaysia; ^4^Biology Division, Center for Foundation Studies in Science, University of Malaya, 50603 Kuala Lumpur, Malaysia; ^5^Department of Pharmacology, Faculty of Medicine, University of Malaya, 50603 Kuala Lumpur, Malaysia

## Abstract

*Curcuma zedoaria* also known as *Temu putih* is traditionally used in food preparations and treatment of various ailments including cancer. The cytotoxic activity of hexane, dichloromethane, ethyl acetate, methanol, and the methanol-soxhlet extracts of *Curcuma zedoaria* rhizomes was tested on two human cancer cell lines (Ca Ski and MCF-7) and a noncancer cell line (HUVEC) using MTT assay. Investigation on the chemical components in the hexane and dichloromethane fractions gave 19 compounds, namely, labda-8(17),12 diene-15,16 dial (**1**), dehydrocurdione (**2**), curcumenone (**3**), comosone II (**4**), curcumenol (**5**), procurcumenol (**6**), germacrone (**7**), zerumbone epoxide (**8**), zederone (**9**), 9-isopropylidene-2,6-dimethyl-11-oxatricyclo[6.2.1.0^1,5^]undec-6-en-8-ol (**10**), furanodiene (**11**), germacrone-4,5-epoxide (**12**), calcaratarin A (**13**), isoprocurcumenol (**14**), germacrone-1,10-epoxide (**15**), zerumin A (**16**), curcumanolide A (**17**), curcuzedoalide (**18**), and gweicurculactone (**19**). Compounds (**1**–**19**) were evaluated for their antiproliferative effect using MTT assay against four cancer cell lines (Ca Ski, MCF-7, PC-3, and HT-29). Curcumenone (**3**) and curcumenol (**5**) displayed strong antiproliferative activity (IC_50_ = 8.3 ± 1.0 and 9.3 ± 0.3 *μ*g/mL, resp.) and were found to induce apoptotic cell death on MCF-7 cells using phase contrast and Hoechst 33342/PI double-staining assay. Thus, the present study provides basis for the ethnomedical application of *Curcuma zedoaria* in the treatment of breast cancer.

## 1. Introduction

It is widely reported that more than 35,000 plant species are used for medicinal purposes worldwide. Of these, 1,200 and 2,000 plant species from Peninsular Malaysia and East Malaysia respectively, are used in folklore medicine [1]. One such plant is* Curcuma zedoaria *(Berg.) Rosc. belonging to the Zingiberaceae family and known by the locals as* Temu putih* or* Kunyit putih.* The leaf blades are 80 cm long, usually with a purple-brown flush running along the midrib on both surfaces of the leaf. In the young plants, the rhizomes of* Curcuma zedoaria *are easily confused with those of* Curcuma aeruginosa *and* Curcuma mangga *because both have almost similar yellow color. However, a cross-section of the rhizomes of the mature plants of* Curcuma aeruginosa* is slightly dark purplish whilst* Curcuma mangga* have brighter yellow color [2].* Temu putih* is used by the Malays in the preparation of traditional medicine—consumed either on their own or in mixtures with other plant species. They are also widely consumed as spices, as flavors in native dishes, and as food preparations in postpartum confinement [2–4].* Curcuma zedoaria *also called* Er-chu* in Chinese is clinically used for the treatment of cervical cancer [5]. In Japan, it has also been used as an aromatic stomachic [6]. Whilst in the Ayurvedic medicine, it is used for the treatment of fevers (cooling), antiseptic, mild expectorant, and deodorizer [7]. In Indonesia,* Curcuma zedoaria* is widely consumed in the form of “jamu” for the treatment of breast and cervical cancers [8]. Medicinal plants are used widely especially in Asia as an alternative medicine for cancer-related diseases because it is believed for having active natural occurring compounds in killing cancer [9, 10]. However, there are only limited studies on the efficacy on the use of medicinal plants [11]. It is therefore important to identify the components which are responsible for the chemotherapeutic effects and the molecular pathway by which these compounds affect cancer cell death. Up to date, there are numerous reported articles on the cytotoxic components of* Curcuma zedoaria* and the mechanism of cell death exerted by some of these compounds [5–8, 12–20]. In our continuous effort to study the bioactive and their mode of actions from medicinal plants of Curcuma species, this communication reports the isolation of 19 compounds and among these, two bioactive compounds (curcumenone and curcumenol) were identified as cytotoxic components and were able to induce apoptosis.

## 2. Materials and Methods

### 2.1. Plant Samples

*Curcuma zedoaria* rhizomes were collected from Tawamangu, Indonesia, and a voucher specimen (KL 5764) was deposited at the herbarium of the Department of Chemistry, Faculty of Science, University of Malaya, Kuala Lumpur, Malaysia.

### 2.2. Extraction of Plant Sample

Briefly, the washed and dried rhizomes of* Curcuma zedoaria* were finely ground. The fine powders of* Curcuma zedoaria* (1.0 kg) were soaked in n-hexane for 3 days. Then, the solvent containing extract was decanted and filtered (were repeated twice each time with five liters of n-hexane). All the filtrates were combined and evaporated using a rotary evaporator (Buchi, Switzerland) to give the n-hexane extract. The n-hexane-insoluble residue was further extracted with CH_2_Cl_2_ to give the CH_2_Cl_2_-soluble extract and CH_2_Cl_2_-insoluble residue. The CH_2_Cl_2_-insoluble residue was further extracted with EtOAc to give the EtOAc-soluble and EtOAc-insoluble extract. The EtOAc-insoluble extract was then extracted with MeOH to give the MeOH extract. The insoluble residue obtained after MeOH extraction was further subjected to soxhlet extraction using methanol to give the MeOH SE extract after evaporation of excess solvent. All the extracts were weighed after solvent evaporation.

### 2.3. Cell Culture

The human cell lines MCF-7 (breast cancer), Ca Ski (cervical cancer), and HT-29 (colon cancer) were cultured as monolayer in RPMI 1640 growth media. HUVEC (human umbilical vein endothelial cells) and PC-3 (prostate cancer) cells were cultured in DMEM. All cells were purchased from the American Tissue Culture Collection (ATCC, USA) except for human umbilical vein endothelial cells (HUVEC) which were obtained from ScienCell Research Laboratories (Carlsbad, CA). All the media were supplemented with 10% v/v foetal bovine serum (FBS), 100 *μ*g/mL penicillin/streptomycin, and 50 *μ*g/mL amphotericin B. The cells were cultured in a 5% CO_2_ incubator at 37°C.

### 2.4. MTT Cytotoxicity Assay

Cell viability was investigated using 3-(4, 5-dimethylthiazol-2-yl)-2, 5-diphenyltetrazolium bromide (MTT) assay. Cells were detached from the 25 cm^3^ tissue culture flask when it achieved 80% confluency. The detached cells were pellet by centrifugation (1,000 rpm; 5 minutes). Cells (3.0 × 10^4^ cells/mL) were seeded onto a 96-well microtiter plate (Nunc). The cells were incubated at 37°C CO_2_ incubator for 24 h to give adherent cells. The test compounds (1–100 *μ*g/mL) were added onto the 96-well microtiter plate containing adherent cells. The untreated cells were incubated in 10% media containing 0.5% DMSO (without addition of any test compounds/extracts). This mixture was regarded as the negative control whereas doxorubicin as the positive control. The plates were incubated for 72 h at 37°C in a 5% CO_2_ incubator. After 72 h, the media were removed and 100 *μ*L of fresh medium and 20 *μ*L of MTT (Sigma, filter sterile, 5 mg/mL) were added to each well and further incubated for 4 hours (37°C) after which the media were substituted with 150 *μ*L DMSO. The 96-well microtiter plates were then agitated at room temperature onto an incubator shaker to dissolve the formazan crystals. The absorbance (*A*) of the content of the plates was measured at 540 nm using a microplate reader. The percentage of inhibition of each test sample was calculated according to the following formula: Percentage of inhibition (%) = (*A*_control_ − *A*_sample_)/*A*_control_ × 100%. The average of three replicates was then obtained. The IC_50_ for each extract was extrapolated from the graphs of the percentage inhibition versus concentration of test agents. Cytotoxicity of each test agent is expressed as IC_50_ value. The IC_50_ value is the concentration of test agents that cause 50% inhibition or cell death, averaged from the three experiments [21–23]. The selectivity index (SI) was also calculated as described by [24, 25] using the ratio between IC_50_ of the extract or compounds on normal cell lines (HUVEC) and IC_50_ of the tested extract or compounds on cancerous cell lines. Selectivity index (SI) values equal or greater than three were considered to have a high selectivity towards cancerous cells. An SI value denotes the selectivity of the sample to the tested cell lines [24, 25].

### 2.5. General Methods on Characterization of the Active Principles

TLC techniques were used to monitor the purity of isolated compounds. Analytical TLC was performed on the precoated plates with silica gel 60 F_254_ (Merck of 20.25 mm) (normal phase). HPTLC and PTLC were carried out on the precoated plates with silica gel 60 GF_254_ (Merck, 20.25 mm). Spots were detected by UV (254, 360 nm) and by spraying of vanillin-H_2_SO_4_ or anisaldehyde-H_2_SO_4_ followed by gentle heating. CC was carried out on Kieselgel 60 (0.043–0.063 mm and 0.063–0.200 mm) (Merck) and Sephadex LH 20 (25–100 m) (Merck). HPLC was used to isolate and purify the compounds. HPLC was performed using Waters System equipped with Binary Gradient Module (Waters 2545), System Fluidics Organizer and Photodiode Array Detector (190–400 nm; Waters 2998), and Sample Manager (Waters 2767). The column used was Waters XBridge Prep C18 5 *μ*M (10 × 250 mm) column with Waters XBridge Prep C18 5 *μ*M (10 × 10 mm) column guard cartridge. The data were collected and analyzed by MassLynx software. 1D NMR (^1^H, ^13^C, Dept 135) and 2D NMR (HSQC, HMBC, COSY, NOESY) spectra were recorded from a JEOL 400 MHz FT NMR spectrometer at 400 MHz for ^1^H-NMR and at 100 MHz for ^13^C-NMR. Chemical shifts in ppm were referenced to the internal standard TMS (*δ* = 0 ppm) for use in ^1^H-NMR and CDCl_3_ (*δ*: 77.0 ppm), ^13^C-NMR spectra, respectively. The GC-MS analyses were performed using Shimadzu QP2010 Series gas chromatography and operated in the split less mode at 275°C. The column used was DM 5MS (5% diphenyl/95% dimethyl polysiloxane) capillary column (30.0 m × 0.25 mm × 0.25 *μ*m) with helium as carrier gas at a flow rate of 1 mL min^−1^. The column temperature was programmed as follows: initially at 60°C, then increased to 250°C at 5°C per minute, and then held for 1 minute. The total ion chromatogram was obtained by autointegration using Chem Station and the components were identified by comparing their mass spectral data with the accompanying Spectral Database (NIST 05, Mass Spectral Library, USA) whenever possible. IR spectra were obtained on a Perkin Elmer 1600 Series FT-IR infrared spectrophotometer with chloroform as solvent. The wavelength is indicated in cm^−1^. Mass spectra of LC-MS were recorded using Agilent Technologies 6530 Accurate-Mass Q-TOF LC-MS.

### 2.6. Extraction and Isolation of Pure Compounds

**Figure 1:**
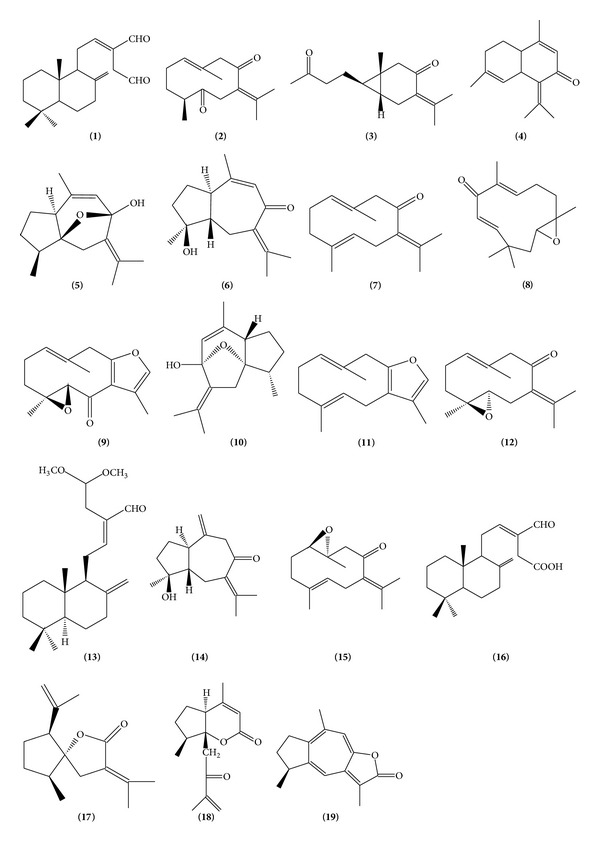
The structures of isolated compounds.

**Figure 2:**
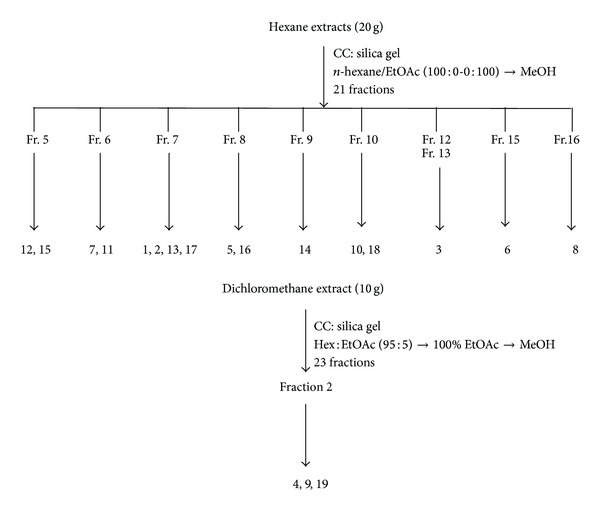
Schematic represents the isolation method of the bioactive compounds from* Curcuma zedoaria*.

The powdered rhizomes (1.0 kg) were initially extracted with hexane to give the hexane extract (24.2 g, 2.4%). The hexane extract (20.0 g) was then subjected to silica gel column chromatography (CC) eluting initially with hexane followed by hexane enriched with increasing percentages of ethyl acetate (EtOAc). Fractions were then combined according to similarity of thin layer chromatography (TLC) spots to give 21 fractions (fractions 1–21). Germacrone-4, 5 epoxide (**12**, 12.4 mg) and germacrone-1, 10 epoxide (**15**, 8.0 mg) were isolated from fraction 5 through micro CC and preparative thin layer chromatography (PTLC). Fraction 6 afforded germacrone (**7**, 21.6 mg) and furanodiene (**11**, 8.8 mg) upon purification with CC and PTLC. Fraction 7 was further chromatographed using various isolation techniques such as Sephadex-LH20, PTLC, and high performance thin layer chromatography (HPTLC) to afford dehydrocurdione (**2**, 34.5 mg), curcumanolide A (**17**, 4.9 mg), and two labdanes, namely, labda-8 (17), 12 diene-15, 16 dial (**1**, 16.2 mg) and labda-8(17), 12 diene-15, 15-dimethoxy-16-al or calcaratarin A (**13**, 22.6 mg). Fraction 8 was further purified using PTLC to give curcumenol (**5**, 15.5 mg) and zerumin A (**16**, 9.8 mg). Isoprocurcumenol (**14**, 10.2 mg) was isolated from fraction 9 using two successive PTLC. Fraction 10 was chromatographed and further purified by HPTLC to afford a second monoclinic modification of curcumenol as a crystallized dimer elucidated by single crystal X-ray diffraction analysis. This dimer is 9-isopropylidene-2, 6-dimethyl-11-oxatricyclo [6.2.1.0^1,5^] undec-6-en-8-ol (**10**, 5.4 mg) as previously described [20], whilst curcuzedoalide (**18**, 13.4 mg) was isolated from fraction 10 using HPLC. Curcumenone (**3**, 16.4 mg) was purified from fractions 12 and 13. Procurcumenol (**6**, 8.9 mg) and zerumbone epoxide (**8**, 11.9 mg) were isolated from fractions 15 and 16, respectively, using micro CC and PTLC. The CH_2_Cl_2_ extract (10 g) was then subjected to silica gel CC with initial elution of 5% EtOAc-hexane and gradually increasing the polarity to 100% EtOAc and finally with MeOH. Fractions were then combined according to similarity of TLC spots to give 23 fractions (fractions 1–23). Fraction 2 was subjected to micro CC to afford comosone II (**4**, 6.6 mg), zederone (**9**, 24.4 mg), and gweicurculactone (**19**, 3.6 mg) upon purification with HPLC. All the isolated compounds were identified using NMR spectroscopy and other supportive data (MS, IR, and UV) and results obtained were consistent with reported data [14–18, 20, 23, 26–32]. The structures of isolated compounds are shown in Figure 1. The method of isolation is summarized in Figure 2.

### 2.7. Phase Contrast Microscopy

Briefly, MCF-7 cells (5 × 10^5^) were grown in a tissue culture dishes (60 mm) for overnight. Then, the cells were treated with curcumenone (**3**) and curcumenol (**5**) at a concentration of 12.5 and 25 *μ*g/mL, respectively. After 48 hours, cells were gently rinsed with PBS. The observation of morphological changes of apoptotic MCF-7 cells after treatment with the two bioactive compounds was viewed using an inverted phase contrast microscope (Leica DMI 3000B, Germany) at 400x magnification according to the method [33].

### 2.8. Fluorescence Microscopy

The morphological features of MCF-7 cells upon treatment by the test compounds also observed by double staining of Hoechst 33342/PI assay [33–37] using the inverted fluorescence microscope (Leica, DM16000B). Briefly, 5 × 10^5^ cells were grown overnight and treated with 12.5 and 25 *μ*g/mL of curcumenone (**3**) and curcumenol (**5**). After treatment of cells with the test compounds for 48 hours, both floating and adherent cells were collected by centrifugation and washed once with cold PBS. Then, Hoechst 33342 solutions (10 *μ*g/mL) were added and incubated at 37°C for 7 minutes. The cells were then stained with PI (2.5 *μ*g/mL) and further incubated in the dark for 15 minutes. Cell suspension (100 *μ*L) was mounted onto glass microscope slides and observed under fluorescence microscope using UV/488 dual excitation (460 nm emission of Hoechst 33342, 575 nm emission of PI). Approximately a total of 200 target cells were calculated and the morphological characteristics of the nuclei were analyzed for quantification of apoptosis and necrosis [37]. The percentage of apoptotic, necrotic, and dead cells was determined according to the formula described by [37].

### 2.9. Statistical Analysis

All data were presented as mean ± standard deviation. All experiments were conducted in triplicates. The data were subjected to one-way analysis of variance (ANOVA) with the significant differences between groups determined by Duncan's multiple range tests (DMRT) at 95% significant difference (*P* < 0.05) using STATGRAPHICS Plus software (version 3.0, Statistical Graphics Corp., Princeton, NJ, USA).

## 3. Results and Discussion

### 3.1. Detection of Cell Viability by MTT Assay

The antiproliferative activity of crude and extracts of* Curcuma zedoaria* was analysed using MTT assay. The IC_50_ values (*μ*g/mL) were evaluated for these crude extracts averaged from three experiments against two human cancer cell lines (Ca Ski and MCF-7) and a noncancer cell (HUVEC) and the result is summarized in Table 1. A plant extract with IC_50_  ≤  20 *μ*g/mL is considered active [21–23]. The hexane extract showed high inhibitory activity against Ca Ski and MCF-7 cells, whilst, the dichloromethane (CH_2_Cl_2_) extract possessed mild cytotoxicity against MCF-7 and exhibited weak cytotoxicity against Ca Ski. The extracts of* Curcuma zedoaria* altogether showed to be essentially ineffective on the normal cells. Selectivity indexes (SI) of the antiproliferative activity of* Curcuma zedoaria* extracts were evaluated by the ratio of the cytotoxic activity (IC_50_) of each extracts against the cancer cells with the normal cells (HUVEC).

The SI with greater or equal value of three was considered to be highly selective towards cancer cells [24, 25, 38, 39]. As shown in Table 1, the hexane extract showed selective activity towards Ca Ski and MCF-7 cells with SI values of 5.3 and 5.4, respectively. Thus, the data have revealed that the hexane extracts exhibited antiproliferative effect and possessed selective activity towards Ca Ski and MCF-7 cells, in reference to normal cells (HUVEC).

### 3.2. Isolation of Active Principles

The isolation of the active principles (compounds** 1–19**) has been described extensively in section methodology. These chemical components were identified using spectroscopic (NMR, IR, and UV) and spectrometric studies (GS-MS, LC-MS, and MS) and were found to be in agreement with reported data [14–18, 20, 23, 26–32].

### 3.3. Antiproliferative Activity of Compounds (**1**–**19**)

The compounds (**1–19**) isolated from the hexane and dichloromethane extracts were further evaluated on four selected cancer cell lines (MCF-7, Ca Ski, HT-29, and PC-3) and a normal human umbilical vein endothelial cell (HUVEC). Many researchers have utilized HUVEC cell lines in determining cytotoxicity of test samples against normal cells [40–44]. Similar approach has also widely employed in high-throughput screening in drug discovery [45]. Test samples showing mild or no toxicity towards normal cell lines (HUVEC) would be a potentially good candidate for drug development. The antiproliferative activity of the compounds (**1–19**) is presented in Table 2. The isolated terpenoids from* Curcuma zedoaria* were found to possess moderate antiproliferative effect against the four selected human carcinoma. In the present study, only curcumenone (**3**) and curcumenol (**5**) demonstrated strong antiproliferative activity against MCF-7. Curcumenone (**3**) was selectively toxic to MCF-7 cells whilst curcumenol (**5**) displayed appreciable selectivity towards MCF-7 in reference to HUVECs with SI values of 6.0 and 2.8, respectively. It appeared therefore that compounds (**3**) and (**5**) have selective activity towards MCF-7 cell line. To the best of our knowledge, there is no report on the cytotoxicity of compounds (**3**), (**5**), and (**10**) against human MCF-7, Ca Ski, and PC-3 cell lines. In a study by [14], curcumenone isolated from the rhizomes of* Curcuma zedoaria* has been reported to have protective effect on alcohol-treated mice and acceleration of liver alcohol dehydrogenase activity. It is also been found as an effective protective effect on D-galactosamine/lipopolysaccharide-induced acute liver injury [5]. In a previous reported publication, [46] claimed that curcumenol is widely used to treat cancer and inflammation and also known as an antibiotic or anticancer drug. In their study, [46] found that curcumenol is not a mechanism-based inhibitor through time- and NADPH-dependent inhibitions and also suggested that curcumenol may be safely used without inducing metabolic drug-drug interaction through P450 inhibition. Labda-8(17), 12 diene-15, 16 dial (**1**) displayed high selective activity towards Ca Ski and appreciable selectivity to MCF-7 but only exhibited moderate cytotoxicity against the cancer cells. Procurcumenol (**6**) and zerumbone epoxide (**8**) exhibited good cytotoxic effect against PC-3 and HT-29 cell lines but were not selective on these tumor cells. Previously, [26] described that zerumbone epoxide isolated from the rhizomes of* Curcuma zedoaria* possessed cytotoxic effects. Thus, this is in a good agreement with our current study. Compounds (**1–10**) displayed appreciable to weak cytotoxic activity against Ca Ski (IC_50_ values ranging from 14.5 ± 0.1 to 100.0 *μ*g/mL, respectively). Only curcumenone (**3**) did not show any antiproliferative activity against Ca Ski. Other compounds (**1**,** 2**,** 5**,** 9**,** 16**, and** 17**) also exhibited moderate inhibitory activity against the tested carcinoma PC-3 and HT-29 cell lines. All tested compounds showed mild cytotoxicity towards the normal cell lines (HUVEC). Curcumenone (**3**) and zerumbone epoxide (**8**) were found to have slight toxicity towards the normal cell. Although the pure compounds are not as effective as doxorubicin in inhibiting the proliferation of the cancer cells, they inflict less damage to the noncancerous cells. To the best of our knowledge, it is important to note that compounds (**1**,** 8**,** 12-13,** and** 19**) are reported here for the first time from* Curcuma zedoaria*. In this study, zerumin A (**16**) was isolated from the hexane fraction and displayed moderate cytotoxic effect on MCF-7, Ca Ski, and PC-3 cell lines. This is in agreement with that reported by [23] whereby zerumin A isolated from* Curcuma mangga *exhibited antiproliferative effect on Ca Ski and MCF-7 displaying IC_50_ of 8.7 ± 0.29 and 14.2 ± 0.06 *μ*g/mL, respectively. In our previous study [19], curzerenone and alismol were also reported present in* Curcuma zedoaria* which were not found in the present study. The reason for this difference is possibly due to the source of the plant samples. The plant sample in the present study was obtained from Tawamangu, Java, Indonesia, whilst those in the earlier report were collected from Jogjakarta, Indonesia. In addition, it is also important to note that diterpenoids (compounds** 1**,** 13**, and** 16**) were not detected in the sample obtained from Jogjakarta, and the isolation of curzerenone and alismol were based on bioassay-guided procedure. The cytotoxicity assay used in the present study could only provide important preliminary data to help select isolated compounds with potential anticancer properties. Further studies on the effect of curcumenone (**3**) and curcumenol (**5**) on the mode of MCF-7 cell death were thus pursued.

### 3.4. Induction of Apoptosis

The result from the cytotoxicity assay provides important preliminary data that may help select compounds with promising anticancer effects for further work. A detailed investigation on the underlying mechanism involved in cell death would provide a more convincing evidence of anticancer effect. Thus, apoptosis induction of the active compounds in the cancer cells was investigated. Apoptosis is described as programmed cell death. It is an essential process that enables the removal of cells from tissues thus maintaining the proper function of multicellular organisms. In the average human adult, about 50–70 billion cells die by apoptosis each day. However, diseases such as cancer resulted when cells fail to die. A series of events is involved in the process of apoptosis. The events start with cell dehydration which leads to cytoplasm condensation and alteration in cell shape and size. The next event is chromatin condensation which starts at the nuclear periphery and results in the concave shape of the nucleus, followed by nuclear membrane integration and nuclear fragmentation. Nuclear fragmentation and other organelles of the apoptotic cells are enveloped by fragments of cytoplasm and form apoptotic bodies which are phagocytosized by neighboring cells, thus preventing inflammatory reaction. These events can be observed in an inverted phase contrast and fluorescence microscope. Thus, apoptosis was first and is still best described morphologically [37]. Induction of apoptosis based on biochemical changes or flow cytometric analyses should always be backed up with morphological studies [37].

### 3.5. Morphology of MCF-7 Cells Treated with Curcumenone and Curcumenol as Observed under Inverted Phase Contrast Microscope

**Figure 3:**
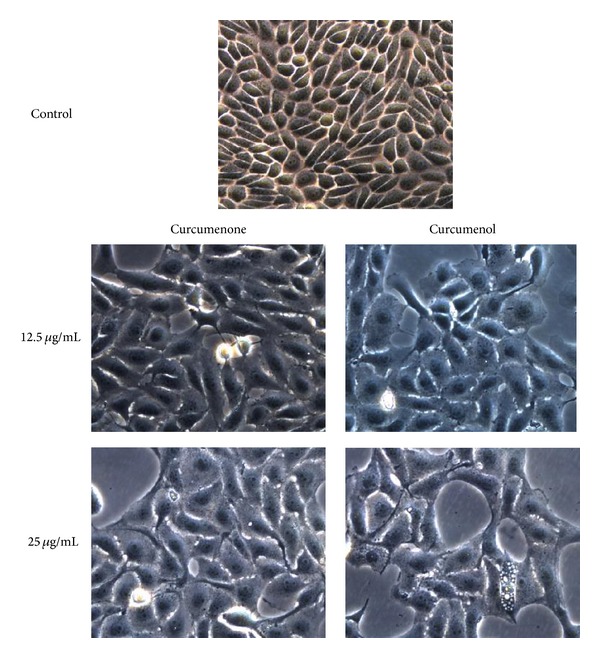
Morphological analysis of MCF-7 cells treated with curcumenone and curcumenol as observed under inverted phase contrast microscope (400x).

In this study, the apoptosis inducing capacity of the two bioactive compounds, namely, curcumenone (**3**) and curcumenol (**5**), on MCF-7 cells was thus investigated using inverted phase contrast microscope. MCF-7 cells were incubated for 48 h with 12.5 and 25 *µ*g/mL of curcumenone (**3**) and curcumenol (**5**), respectively. Exposure of MCF-7 cells to the compounds led to cell shrinkage, loss of contact with adjacent cells, and decrease in cell numbers (Figure 3). In comparison, the untreated (control) cells were observed as intact and were cuboids or polygonal in shape. Floating cells detached from the surface of the tissue culture dishes (not shown) were also observed.

### 3.6. Hoechst 33342/PI Staining of MCF-7 Cells upon Treatment with Curcumenone and Curcumenol

**Figure 4:**
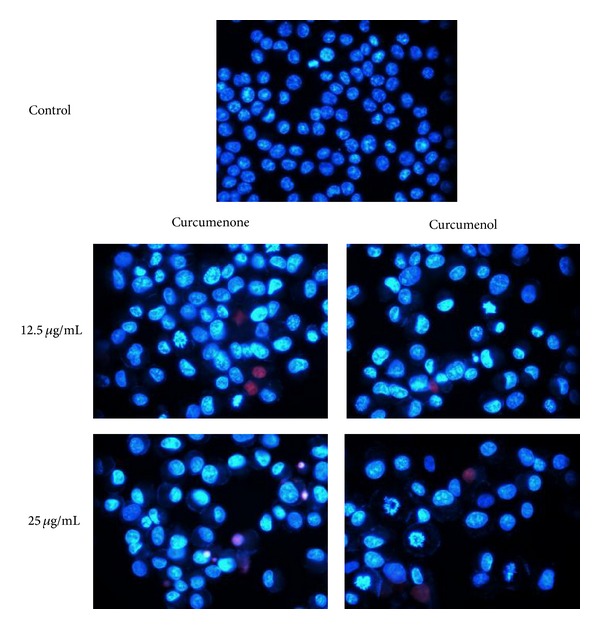
Apoptosis-inducing effect of curcumenone and curcumenol on MCF-7 cells by double staining using Hoechst 33342/PI and visualized under fluorescence microscope (630x).

**Figure 5:**
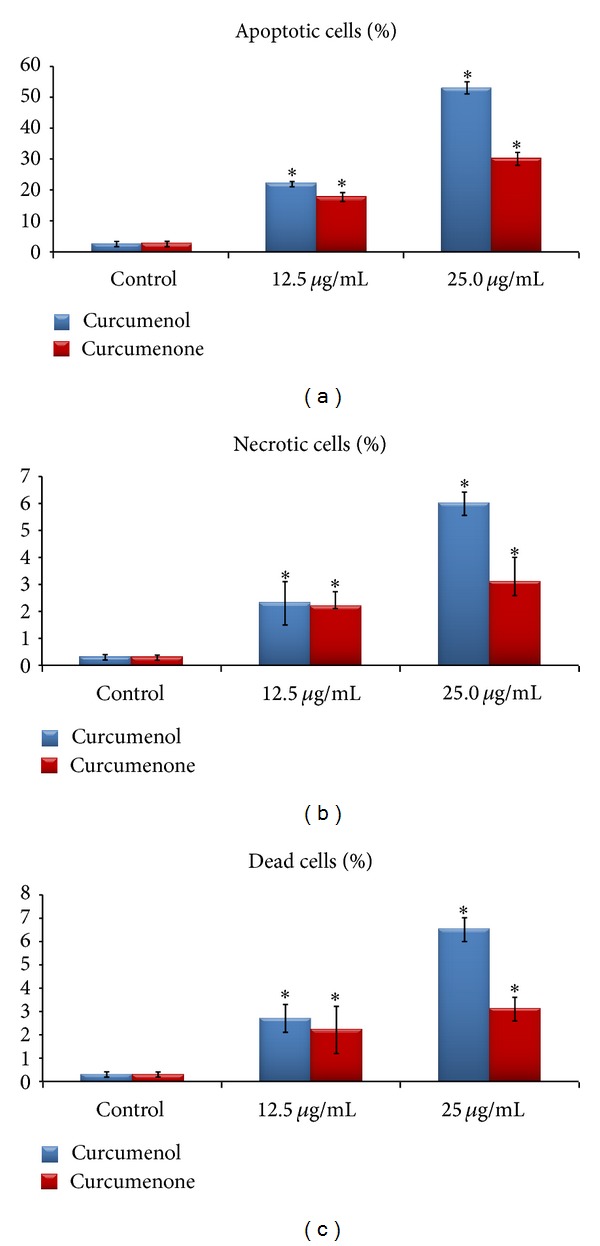
Apoptotic index and percentage of necrotic cells. Values expressed are means ± standard deviation (s.d.) of triplicate measurements. The asterisks (∗) denote significant differences between groups (*P* < 0.05).

Dual staining by Hoechst 33342/propidium iodide (PI) of MCF-7 and Ca Ski cells revealed that induction of apoptotic death occurred after 48 h incubation with curcumenone (**3**) and curcumenol (**5**). The untreated cells displayed intact regular form and were homogenously stained with a dimmer blue color. After the cells were treated (48 h) with 12.5 and 25 *μ*g/mL of curcumenone (**3**) and curcumenol (**5**), respectively, apoptotic nuclei emitted much brighter blue fluorescence due to the highly condensed chromatin. As in Figure 4, crescents were observed around the periphery of the nucleus due to chromatin condensation. Cells that were in late apoptosis emitted pink fluorescence. The organized structure of pink chromatin denoted dead cells with normal nuclei. Dead cells with apoptotic nuclei showed highly condensed and fragmented bright pink chromatin. Necrotic cells were swollen with irregular membranes and fluorescence bright pink chromatin (due to PI). There was a significant increase in the percentage of apoptotic cells due to increasing dose of tested compounds. Curcumenol (**5**) revealed better inducing apoptosis capacity in comparison to curcumenone (**3**) as observed in Figure 5.

## 4. Conclusions

In this study,* Curcuma zedoaria *was shown to possess several compounds that have antiproliferative effect on four cancer cell lines (MCF-7, Ca Ski, PC-3, and HT-29). Amongst these, two compounds, namely, curcumenone (**3**) and curcumenol (**5**), present in the hexane extract were able to induce apoptosis in MCF-7 cells by inhibiting the proliferation of the cancer cells. However, further investigations are necessary to determine their mode of action. It is noteworthy to mention that the hexane extract and the two compounds curcumenone and curcumenol showed low toxicity towards the normal cell line (HUVEC). If this also occurs* in vivo* then this plant has the potential to be developed as anticancer agent.

## Figures and Tables

**Table 1:** Antiproliferative activity [IC_50_ values (*µ*g/mL)] and selectivity index of crude and fractionated extracts of *Curcuma zedoaria *against human cancer and noncancer (HUVEC) cell lines.

Extracts	IC_50_ (*µ*g/mL)^a^				
	MCF-7	SI^b^	Ca Ski	SI^b^	HUVEC
Hexane	18.4 ± 1.6	5.4	19.0 ± 1.5	5.3	>100.0
Dichloromethane	40.6 ± 2.3	2.5	83.5 ± 2.7	1.2	>100.0
Ethyl acetate	>100.0	1.0	>100.0	1.0	>100.0
Methanol	>100.0	1.0	>100.0	1.0	>100.0
Methanol (soxhlet extraction)	>100.0	1.0	>100.0	1.0	>100.0

^a^Data are presented as mean ± standard deviation (SD) of three replicates.

^b^SI is the selectivity index. SI values ≥3.0 denote high selectivity towards cancerous cells.

**Table 2:** Antiproliferative activity [IC_50_ values (*µ*g/mL)]^a^ and selectivity index of isolated compounds against selected human cancer cell lines and human umbilical vein endothelial cells (HUVEC).

Compounds	IC_50_ (*µ*g/mL) SI^b^				
	MCF-7	Ca Ski	PC-3	HT-29	HUVEC
labda-8(17), 12 diene-15, 16 dial (**1**)	16.3 ± 0.2 (2.8)	14.5 ± 0.1 (3.1)	26.3 ± 2.4 (1.7)	21.5 ± 3.1 (2.1)	45.3 ± 1.9
dehydrocurdione (**2**)	33.0 ± 1.1 (0.7)	21.7 ± 1.1 (1.1)	19.1 ± 2.8 (1.3)	22.7 ± 2.4 (1.1)	24.0 ± 2.1
curcumenone (**3**)	8.3 ± 1.0 (6.0)	>100.0 (0.5)	39.8 ± 4.2 (1.3)	43.3 ± 6.2 (1.2)	50.0 ± 8.6
comosone II (**4**)	>100.0	76.0 ± 1.2	na	na	na
curcumenol (**5**)	9.3 ± 0.3 (2.8)	18.5 ± 1.0 (1.4)	17.3 ± 1.2 (1.5)	24.8 ± 2.7 (1.0)	25.9 ± 1.4
procurcumenol (**6**)	16.1 ± 2.2 (1.0)	62.4 ± 0.3 (0.3)	13.3 ± 1.7 (1.2)	15.5 ± 2.3 (1.1)	16.3 ± 1.0
germacrone (**7**)	59.1 ± 2.9 (1.2)	39.3 ± 1.2 (1.9)	55.2 ± 4.9 (1.3)	42.9 ± 4.1 (1.7)	73.7 ± 0.3
zerumbone epoxide (**8**)	24.1 ± 0.1 (0.6)	34.5 ± 0.6 (0.4)	10.8 ± 1.9 (1.3)	13.7 ± 2.7 (1.0)	14.2 ± 1.1
zederone (**9**)	>100.0 (0.4)	>100.0 (0.4)	27.0 ± 1.9 (1.6)	19.1 ± 2.5 (2.2)	42.1 ± 2.7
second monoclinic curcumenol (**10**)	>100.0 (0.7)	>100.0 (0.7)	na	na	71.7 ± 6.1
furanodiene (**11**)	36.5 ± 2.6 (1.1)	na	39.5 ± 4.5 (1.0)	47.2 ± 4.4 (0.9)	40.9 ± 2.6
germacrone-4, 5-epoxide (**12**)	37.2 ± 4.0 (1.3)	na	43.9 ± 7.2 (1.1)	39.6 ± 4.6 (1.2)	48.4 ± 4.7
calcaratarin A (**13**)	62.5 ± 4.8 (0.8)	na	41.7 ± 3.4 (1.1)	48.3 ± 5.1 (1.0)	47.3 ± 4.2
isoprocurcumenol (**14**)	58.8 ± 4.2 (0.8)	na	37.4 ± 4.5 (1.2)	51.6 ± 3.9 (0.9)	45.1 ± 3.0
germacrone-1, 10-epoxide (**15**)	61.2 ± 5.8 (0.9)	na	53.2 ± 4.9 (1.0)	72.8 ± 8.3 (0.8)	55.5 ± 1.6
zerumin A (**16**)	22.3 ± 1.1 (1.2)	na	21.9 ± 1.6 (1.2)	17.4 ± 2.0 (1.5)	25.8 ± 1.9
curcumanolide A (**17**)	29.8 ± 3.1 (0.7)	na	18.8 ± 2.4 (1.2)	21.3 ± 3.2 (1.0)	21.7 ± 7.0
curcuzedoalide (**18**)	49.8 ± 3.6 (0.9)	na	62.1 ± 8.1 (0.7)	58.2 ± 3.5 (0.8)	45.3 ± 7.8
gweicurculactone (**19**)	31.2 ± 3.2 (2.3)	na	38.3 ± 2.2 (1.9)	35.7 ± 5.8	71.7 ± 6.1 (2.0)
doxorubicin∗	0.1 ± 0.0 (4.0)	0.2 ± 1.0 (2.0)	na	na	1.4 ± 0.0

^a^Data are presented as mean ± standard deviation (SD) of three replicates.

^b^SI is the selectivity index. SI ≥3.0 denotes high selectivity towards cancerous cells.

∗na-not available.
